# Significant Risks and Responsibilities in the Clinical Use of AI Predictive Models. Comment on: “AI Predictive Model of Mortality and Intensive Care Unit Admission in the COVID-19 Pandemic: Retrospective Population Cohort Study of 12,000 Patients”

**DOI:** 10.2196/81251

**Published:** 2025-09-11

**Authors:** Aymen Boud, Ishraq Hussein

**Affiliations:** 1 Leicester Medical School University of Leicester Leicester United Kingdom

**Keywords:** artificial intelligence, machine learning, clinical decision support, patient safety, health care ethics, SARS-CoV-2, COVID-19, death, mortality, intensive care unit, population study, predictive model, random forest

The retrospective study by Ruiz Giardin et al [1], “AI Predictive Model of Mortality and Intensive Care Unit Admission in the COVID-19 Pandemic: Retrospective Population Cohort Study of 12,000 Patients,” provided valuable insights into the application of random forest (RF), a supervised machine learning (ML) technique, for predicting patient mortality and intensive care unit admission during the COVID-19 pandemic. We thank the authors for their contribution. However, there are concerns about artificial intelligence (AI)–driven overestimation, particularly in relation to patient safety and the documentation of AI outputs in patient records.

The RF model used in the study derives outcome probabilities, resulting in a “yes” for mortality when a predefined threshold is met. While 98% to 99% of patients predicted to survive did so, in contrast, only 21% to 25% of those predicted to die actually did [1]. This notably low positive predictive value, combined with the “black box” nature of ML models, which lack transparent explanations of outputs [2], raises questions for future use of the RF model in hospital triaging. Shapley additive explanations were used to identify which patient factors most influenced predictions, but this information may not be readily available to health care staff [1]. Although patient data were anonymized for the study, introducing AI-generated outputs into patient records could have consequences for health care management. Such outputs could inadvertently lead to overly intensive treatments stemming from AI overestimations. This poses a risk of physical and psychological harm, especially if patients later discover their care was based on inaccurate estimations.

While we recognize the upside of AI tools like RF in health care, they should not serve as a replacement for clinicians. Clinicians have a clear duty to protect patient well-being and maintain their trust in health care systems. Therefore, the development of patient safeguarding measures and guidelines for the clinical use of AI is essential, and we hope the authors will take this into account. Future integration of models such as the RF model into other health care systems requires careful consideration, particularly in private systems such as those in the United States. Life, disability, and long-term care insurers are exempt from the Health Insurance Portability and Accountability Act (HIPAA) and can lawfully request full medical records during underwriting, with patient consent [3]. If AI-generated outputs are included in patient records, they may influence long-term health care management by affecting insurance coverage decisions and premiums. Given that 36% of uninsured disabled Americans already struggle to access care [4], AI could further disadvantage vulnerable patients.

To conclude, we urge the authors to address how AI outputs are documented and interpreted to protect patient safety and confidentiality. It is equally important to consider developing guidelines for the use of AI tools and ML models such as the RF model, ensuring these tools support clinicians in their diagnostic processes, not replace their clinical judgment. Addressing these considerations is essential to enable such AI tools to improve patient outcomes without introducing unintended risks.

## Abbreviations

AI: artificial intelligence
HIPAA: Health Insurance Portability and Accountability Act
ML: machine learning
RF: random forest
